# Clinical Profiles of Selected Biomarkers Identifying Infection and Cancer Patients: A Gorzów Hospital Example

**DOI:** 10.1155/2019/6826127

**Published:** 2019-09-02

**Authors:** Katarzyna Brzeźniakiewicz-Janus, Marcus Daniel Lancé, Andrzej Tukiendorf, Mirosław Franków, Joanna Rupa-Matysek, Edyta Wolny-Rokicka, Lidia Gil

**Affiliations:** ^1^Department of Hematology, Multi-Specialist Hospital Gorzów Wielkopolski, Faculty of Medicine and Health Science, University of Zielona Góra, Gorzów Wielkopolski, Poland; ^2^Department of Anesthesiology, Intensive Care Unit and Perioperative Medicine, Hamad Medical Corporation, Doha, Qatar; ^3^Department of Public Health, Wrocław Medical University, Wrocław, Poland; ^4^Department of Hematology and Bone Marrow Transplantation, Poznań University of Medical Sciences, Poznań, Poland; ^5^Department of Surgery and Oncology, Multi-Specialist Hospital Gorzów Wielkopolski, Faculty of Medicine and Health Science, University of Zielona Góra, Gorzów Wielkopolski, Poland

## Abstract

**Introduction:**

Many pathobiological processes that manifest in a patient's organs could be associated with biomarker levels that are detectable in different human systems. However, biomarkers that promote early disease diagnosis should not be tested only in personalized medicine but also in large-scale diagnostic evaluations of patients, such as for medical management.

**Objective:**

We aimed to create an easy algorithmic risk assessment tool that is based on obtainable “everyday” biomarkers, identifying infection and cancer patients.

**Patients:**

We obtained the study data from the electronic medical records of 517 patients (186 infection and 331 cancer episodes) hospitalized at Gorzów Hospital, Poland, over a one and a half-year period from the 1^st^ of January 2017 to the 30^th^ of June 2018.

**Methods and Results:**

A set of consecutive statistical methods (cluster analysis, ANOVA, and ROC analysis) was used to predict infection and cancer. For in-hospital diagnosis, our approach showed independent clusters of patients by age, sex, MPV, and disease fractions. From the set of available “everyday” biomarkers, we established the most likely bioindicators for infection and cancer together with their classification cutoffs.

**Conclusions:**

Despite infection and cancer being very different diseases in their clinical characteristics, it seems possible to discriminate them using “everyday” biomarkers and popular statistical methods. The estimated cutoffs for the specified biomarkers can be used to allocate patients to appropriate risk groups for stratification purposes (medical management or epidemiological administration).

## 1. Introduction

Many pathobiological processes that manifest in a patient's organs could be associated with biomarker levels that are detectable in different human systems (e.g., reactive oxygen species in the systemic circulation [1] or apolipoprotein E in Alzheimer's disease [2]). The accessibility of biomarkers that promote early disease diagnosis in both symptomatic and asymptomatic patients, such as infection and cancer, could lead to the application of personalized medicine in many serious diseases. However, a biomarker should be tested in epidemiological diagnostics for its qualifications. An ideal biomarker should be reliable objectively distinguishing between normal biologic and pathological processes (regardless of methodological difficulties and expenses). In a large-scale diagnostic evaluation of patients for medical management, for example, a biomarker should also be standardized (accurately measured in different laboratories), timely (not time-consuming), practical (comprehensible), and inexpensive (cost-effective) [2].

There has been increasing interest in identifying disease-related biomarkers to predict pathogenic processes. However, there is still minimal information about how biomarkers relate to disease progression, severity, or response to therapy.

For instance, in cardiovascular disease, it remains uncertain whether the association between low vitamin D status and arterial disease is causal or whether it is just a side factor. The results of the study in [3] demonstrated that low vitamin D status was a risk factor for the severity of arterial disease. Similarly, triglyceride-cholesterol (TG-C) imbalance across lipoprotein subclasses predicts diabetic kidney disease and mortality in type 1 diabetes. In this report, low C, low TG-C ratio, and high TG-C ratio subclasses represented three phenotypes associated with increasing patient mortality (<3%, 6%, and 40%, respectively) [4]. Long-term total and cardiovascular mortality in patients undergoing coronary angiography was associated with mean LDL particle diameter (large >16.8 nm, intermediate 16.5–16.8 nm, and small <16.5 nm) [5]. The study authors conclude that both large and small LDL diameters were independently associated with increased risk mortality of all causes compared with LDL of intermediate size [5]. However, circulating lipids were also associated with alcoholic liver cirrhosis and represented potential biomarkers for risk assessment [6]. In this study, 6 of the 25 lipid classes and subclasses were significantly associated with alcoholic liver cirrhosis. Based on the multivariate classification models, the authors established that the addition of lipid measurements to the clinical characteristics of patients resulted in improved ability to estimate the severity of liver cirrhosis [6]. These data were confirmed by [7], who suggested that metabolomic profiles based on diacyl-phosphatidylcholines, lysophosphatidylcholines, ether lipids, and sphingolipids were a new class of biomarkers for excess alcohol intake and had potential for future epidemiological and clinical studies. A study of viral haemorrhagic fever [8] highlights that the most common clinical and laboratory profiles are very helpful for diagnosis of dengue viral infections. In the population of 102 dengue patients, elevated levels of AST in 46 (45.1%) and ALT in 18 (17.6%) patients were found to be among the most common clinical manifestations of the infection. This finding could alert physicians to the likelihood of dengue virus infections in the study area. In other words, a single biomarker could be indicative of several pathologies that do not necessarily have a direct relationship. Depending on the combinations of markers, different diseases could be detected by the biomarkers.

In this paper, we show how a combined statistical methodology based on “everyday” diagnostic investigations contributes to differentiating infection from cancer patients at an early stage. With our blinded data analysis approach, a new diagnostic filter could easily categorize the patients into appropriate disease categories. We used infection and cancer patients following a priori clinical assumptions that these diseases are very different in their clinical characteristics. For this reason, we believe they will contribute to more reliable cutoffs of the possible biomarkers to distinguish early symptoms, and for stratification with a few biomarkers, the chance of classifying patients into the wrong category will be diminished.

## 2. Materials

Altogether, 517 individual MPV measurements, one for each patient (F = 55%, aged = 53.4 ± 27.3, ranged 1–96), were available for the 186 diagnosed with infection (36%) and 331 (64%) cancer (new and old) diagnoses at the Multi-Specialist Hospital Gorzów Wielkopolski from the 1^st^ of January 2017 to the 30^th^ of June 2018.

Measurements of the blood biomarkers were performed in the hospital laboratory unit using a Sysmex XN-2000 (Sysmex Corporation, Japan) analytical system with EDTA-KE/2.7 mL samples. Additionally, serum Z/4.9 mL samples were used to measure CRP. The descriptive statistics for MPV and other selected morphological biomarkers are given in Table 1.

The Bioethical Committee of Poznań University of Medical Sciences approved the study, and it was conducted in accordance with the Declaration of Helsinki (No. KB-1028/17).

At this point, we realized that the use of retrospective data may be the most concerning limitation of our study. Although the data used in the analysis were mostly complete (*n* = 517), some unorganized or incomplete medical records could bias inferential statistics, especially for neutrophils, lymphocytes, and monocytes.

## 3. Methods and Results

The computation was performed with the R statistical platform [9].

To avoid highly multidimensional data analysis due to examination of several biomarkers at the same time, our investigation of the clinical profiles of the diseases, we started from the mean platelet volume (MPV), a marker derived from common whole blood count. The choice of the MPV biomarker was justified by the fact that the immune system is linked to platelet function because platelets are involved in the so-called innate immunity [10]. Platelet count and MPV could be indicative of an infection. On the other side, cancer is a consumptive disease that frequently affects bone marrow function by suppressing it. In addition, treating cancer with chemotherapy induces bone marrow depression in all cell lines, which finally results in altered platelet parameters.

**Figure 1:**
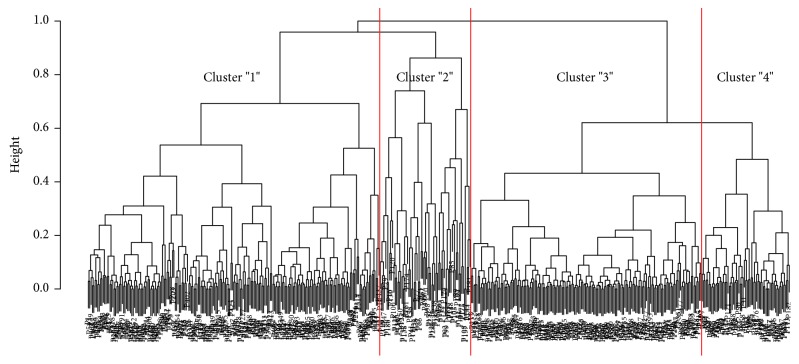
Classification tree (dendrogram) of patients.

In the statistical analysis, a cluster analysis was first performed using the simple idea described by [11]. Based on patient age and sex and their MPV, a classification tree (dendrogram) of patients was created [12] and is presented in Figure 1.

Description: in Figure 1, particular patients (coded by “P” numbers) from up to down are hierarchically aggregated in separated branches that are represented by individual leaves of the dendrogram. Four main families (clusters “1,” “2,” “3,” and “4”) of patients can be distinguished in the created classification tree based on patient age and sex and their MPV.

Following the “P” numbers, the identification of patients was performed, and descriptive statistics of the established clusters were conducted using one-way ANOVAs with adjusted Bonferroni-corrected *p* values (Table 2).

**Figure 2:**
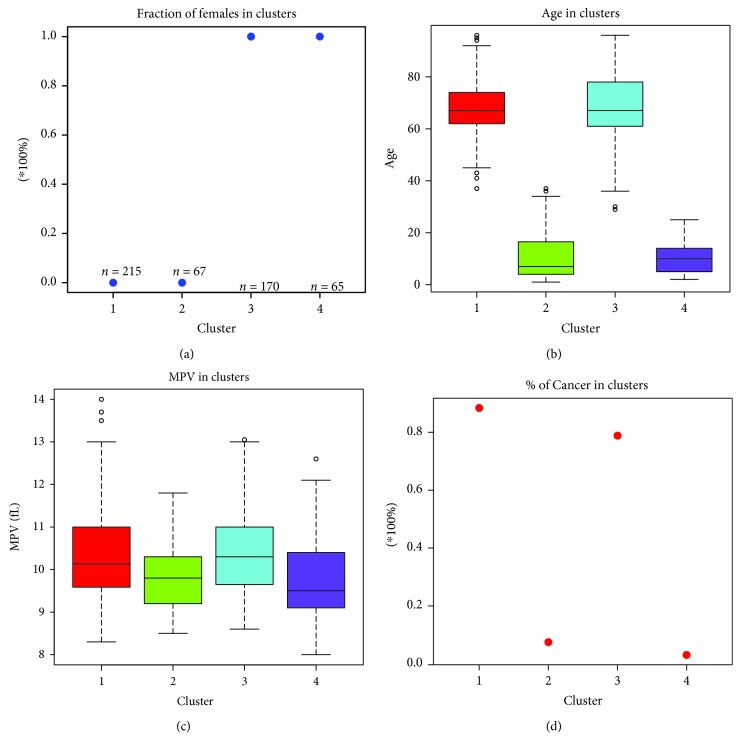
Fraction plot of clusters of patients by sex (a), age (b), MPV (c), and cancer fraction (d).

From the results reported in Table 2, we can see the sex separation between the clusters (1 and 2 = males and 3 and 4 = females) and the estimated *p* values indicate highly representative clusters of patients. The plots of means for the analysed risk factors (age, sex, MPV, and cancer) are presented in the corresponding Figure 2.

To complete the one-way ANOVAs, multiple comparisons between clusters (post hoc Tukey's HSD and Dunn's test *p* values) for age and MPV were performed (Table 3).

It can be seen in Table 3 that clusters 1 and 3 and 2 and 4 are nearly “identical” in terms of the analysed clinical factors (age, MPV, and cancer). For the remaining biomarkers, additional one-way ANOVAs were conducted (with Bonferroni adjustment) to check for differences among the clusters (Table 4).

**Figure 3:**
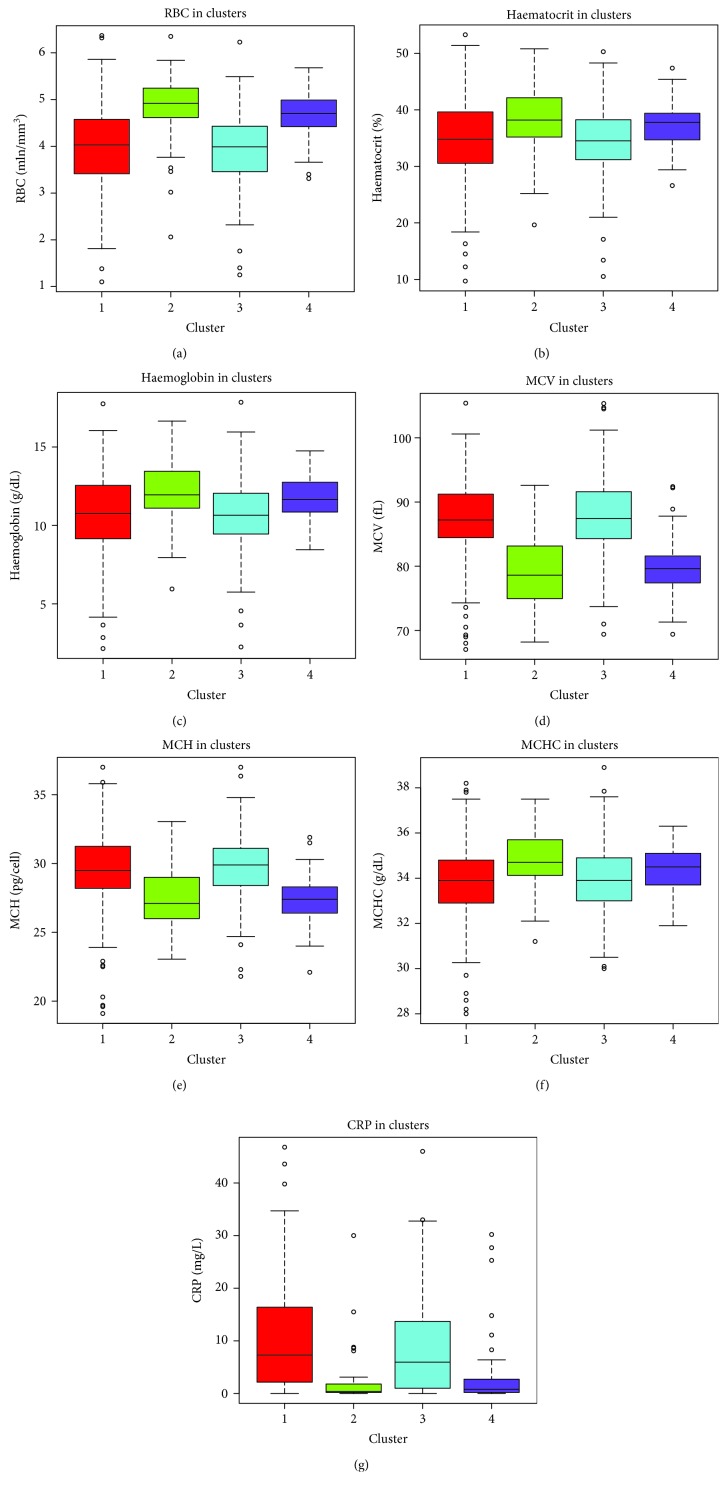
Plot of mean WBC results in clusters of patients: (a) red blood cells, (b) haematocrit, (c) haemoglobin, (d) mean corpuscular volume, (e) mean corpuscular haemoglobin, (f) mean corpuscular haemoglobin concentration, and (g) C-reactive protein.

From the statistical estimates reported in Table 4, we can see that the diseases (infection and cancer) and the seven selected biomarkers (RBC, haematocrit, haemoglobin, MCV, MCH, MCHC, and CRP) statistically significantly different among the established clusters of patients. For these results, plots of means are presented in the corresponding Figure 3.

Finally, and separately for males (clusters 3 and 4) and females (clusters 1 and 2), ROC analysis [13] was conducted to estimate the cutoffs for the statistically significant classifiers (biomarkers) predicting infection and cancer in the analysed group of patients (Table 5).

From the results reported in Table 5, we can see that age and all selected biomarkers were statistically significant classifiers of the clusters within male and female patients. The most predictive were age for males (39) and females (27), which segregated patients with 100% precision. Next, MCV had AUCs of 84% and 87%, respectively. MPV in males had the poorest AUC (63%), as did MCHC in females (62%). Based on these values, we can establish profiles of infection and cancer patients with reference to estimated threshold values (Table 6).

## 4. Discussion

In addition to classic solutions, modern statistical techniques are becoming more and more popular in personalized medicine and important for clinical trials. In the study of biomarkers in which regression modelling fails, cluster analysis [14], decision tree analysis [15], principal component analysis [16], network analysis [17], and receiver operating characteristic curves [18] are progressively gaining greater research significance.

The current study shows how application of modern statistical ideas could add to the classical approaches by screening and combining biomarkers distinguishing between different diseases such as infection and cancer. In clinical practice, patients are not diagnosed with biomarkers only but those combined with symptoms, medical history, and imaging (e.g., X-ray and ultrasound). Principally, there should be serious suspicion or signs, such as fever or redness, before blood tests are ordered. However, sometimes the symptoms are not very specific. In that sense, a predefined search strategy could help identify risk factors for disease on an individual basis.

As we assumed, the most incomplete data bioindicators (i.e., neutrophils, lymphocytes, and monocytes) did not play a role in our biomarker analysis. However, we showed that basing on age and the remaining “everyday” biomarkers (i.e., MPV, RBC, haematocrit, haemoglobin, MCV, MCH, MCHC, and CRP) provided statistically reliable cutoffs of their levels for distinguishing early symptoms of the analysed diseases for stratification purposes. After a patient has been allocated to a “risk group,” more specific investigations could be initiated. Taking several successive bioindicators together will improve the precision of the classification. In other words, the more indicators are fed into the analysis, the stronger the results get, which will reduce the probability of misclassification.

At this point, it seems necessary to comment on the classification of patients by their age. It is only the preselection of patients in our study that would cause erroneous allocation of “young cancer” cases and “old infection” patients. However, if the remaining seven bioindicators were more specific, this disadvantage could be overcome. We realize that the validation of patients does not have to be just perfect. Having several biomarkers at our disposal, however, we can determine a possible risk group with high probability.

Such an approach speeds up finding a diagnosis and avoids unnecessary investigations that may afflict patients. Both could save time, increase patient comfort and well-being, and save money by reducing ineffective studies. Additionally, such an epidemiological solution could be an easy and effective tool to place patients on appropriate clinical treatment pathways, which finally could impact economic results. Moreover, this tool could be important after exhausting conventional tests and statistics to predict and diagnose an underlying, unspecified disease for an individual patient. Again, combining a set of markers derived from a cluster search could help identify the correct disease and treatment. For example, the mean platelet volume is a general marker that has been associated with all kinds of diseases. Although there are still some methodological concerns, the strength of MPV as a diagnostic marker may increase in value using modern statistics. As each marker per se is not strong enough to distinguish clusters, it helps to combine these preselected markers and search then for correlations with specific diseases.

At the end of this discussion, we would like to emphasize one more important conclusion coming from our research, i.e., that not only very unique and precise diagnostic devices and tests need to be obligatory for a meaningful assessment of patient health. We can use standard and inexpensive biomarkers as sufficient analytical tools with strong and invaluable diagnostic power.

## 5. Conclusions

Based on the gathered material, the clinical and statistical methods, and the obtained results, the following conclusions can be drawn from our study:

- Despite infection and cancer being very different diseases in their clinical characteristics, it seems possible to discriminate them using “everyday” biomarkers and popular statistical methods
- The estimated cutoffs of the specified biomarkers can be used to allocate patients to the appropriate “risk group” for stratification purposes
- We believe that filtering by a few biomarkers could diminish the chance of classification of patients into the wrong categories of the disease
- The presented methodology can be of use in a large-scale diagnostic evaluation of patients, such as for medical management or epidemiological administration
- Standard diagnostic tests may be sufficient to allocate patients to an increased risk group without the need for unique and expensive analytical methods
- The diagnostic qualification of disease cases may depend on the assumed number of clinical criteria met in the algorithm
- The established clinical norms for the biomarkers should undergo scientific verification and comparison in other populations

## Figures and Tables

**Table 1:** Description statistics of selected biomarkers.

Biomarker	*n*	Mean	SD	Me	Min	Max
MPV (fL)	517	10.2	1.03	10.1	8.0	14.0
WBC (10^9^/L)	515	12.8	12.3	10.5	0.12	200
Neutrophils (10^9^/L)	221	7.81	7.03	6.21	0.01	47.7
Lymphocytes (10^9^/L)	219	3.16	12.6	1.35	0.05	174
Monocytes (10^9^/L)	219	1.19	3.15	0.75	0.01	33.6
RBC (mln/mm^3^)	517	4.16	0.85	4.27	1.10	6.37
Haematocrit (%)	517	35.2	6.5	35.7	9.7	53.3
Haemoglobin (g/dL)	517	12	2.35	12.1	3.2	18.9
MCV (fL)	517	85.4	6.89	85.8	67.0	105
MCH (pg/cell)	517	29.1	2.6	29.2	19.1	37
MCHC (g/dL)	517	34.0	1.51	34.1	28	38.9
PLT (10^9^/L)	517	279	152	260	24	1997
CRP (mg/L)	453	7.56	9.18	3.2	0.01	46.8

**Table 2:** Characteristics of patients in clusters (mean ± st.dev./median (range)) with Bonferroni-adjusted *p* values.

Cluster	*n*	Sex	Age	MPV (fL)	Cancer (%)
1	215	Males	67.8 ± 10/67 (37–96)	10.3 ± 1.05/10.13 (8.3–14)	88.4
2	67	Males	11.2 ± 9.4/7 (1–37)	9.82 ± 0.75/9.8 (8.5-11.8)	7.5
3	170	Females	68.1 ± 14/67 (29–96)	10.4 ± 1.04/10.3 (8.6–13.1)	78.8
4	65	Females	10.4 ± 6.6/10 (2–25)	9.78 ± 0.98/9.5 (8–12.6)	3.1

	*p* value	—	<0.001	<0.001	<0.001

**Table 3:** Multiple comparisons between clusters (post hoc Tukey's HSD and Dunn's test *p* values) for age, MPV, and cancer fraction.

Difference between clusters	Age	MPV (fL)	Cancer (%)
2-1	<0.001	0.003	<0.001
3-1	0.995	0.759	0.106
4-1	<0.001	0.001	<0.001
3-2	<0.001	<0.001	<0.001
4-2	0.977	0.995	0.600
4-3	<0.001	<0.001	<0.001

**Table 4:** Kruskal's test and one-way ANOVAs with Bonferroni adjustment.

Biomarker	*p* value	Bonferroni
WBC (10^9^/L)	0.153	1.000
Neutrophils (10^9^/L)	0.469	1.000
Lymphocytes (10^9^/L)	0.468	1.000
Monocytes (10^9^/L)	0.603	1.000
RBC (mln/mm^3^)	<0.001	<0.001
Haematocrit (%)	<0.001	<0.001
Haemoglobin (g/dL)	<0.001	<0.001
MCV (fL)	<0.001	<0.001
MCH (pg/cell)	<0.001	<0.001
MCHC (g/dL)	<0.001	<0.001
PLT (10^9^/L)	0.949	1.000
CRP (mg/L)	<0.001	<0.001

**Table 5:** ROC analysis of patients (with cutoffs, AUC, and 95% CI).

Sex:	Males			Females		
Clinical factor	Cutoff	AUC	95% CI	Cutoff	AUC	95% CI
Age	39	100%	100%-100%	27	100%	100%-100%
MPV (fL)	10.65	63%	56%-70%	9.55	68%	60%-76%
RBC (mln/mm^3^)	4.50	81%	75%-87%	4.31	80%	74%-86%
Haematocrit (%)	33.5	66%	59%-73%	33.7	65%	58%-72%
Haemoglobin (g/dL)	12.0	69%	63%-76%	11.5	67%	60%-74%
MCV (fL)	84.1	84%	78%-89%	82.3	87%	82%-92%
MCH (pg/cell)	27.2	74%	68%-81%	28.6	80%	74%-86%
MCHC (g/dL)	34.0	70%	63%-77%	33.6	62%	55%-69%
CRP (mg/L)	2.63	84%	78%-89%	4.38	74%	66%-81%

**Table 6:** Clinical profiles of infection and cancer for selected clinical factors.

Clinical factor/disease	Infection	Cancer
Age	Younger	Older
MPV (fL)	Lower	Higher
RBC (mln/mm^3^)	Higher	Lower
Haematocrit (%)	Higher	Lower
Haemoglobin (g/dL)	Higher	Lower
MCV (fL)	Lower	Higher
MCH (pg/cell)	Lower	Higher
MCHC (g/dL)	Higher	Lower
CRP (mg/L)	Lower	Higher

## Data Availability

Personal information on the study patients has been anonymized, and the dataset used in the statistical analysis is completely available for further clinical trials upon request.

## Abbreviations

ALT: Alanine transaminase
ANOVA: Analysis of variance
AST: Aspartate transaminase
CI: Confidence interval
CRP: C-reactive protein
HSD: Honest significant difference
LDL: Low-density lipoprotein
MCH: Mean corpuscular haemoglobin
MCHC: Mean corpuscular haemoglobin concentration
MCV: Mean corpuscular volume
MPV: Mean platelet volume
PLT: Platelets
RBC: Red blood cell
ROC: Receiver operating characteristic
TG-C: Triglyceride-cholesterol
WBC: White blood cell.
